# Impact of creatinine measurement methods on eGFR and GFR category assignment

**DOI:** 10.62838/jccm-2026-0007

**Published:** 2026-04-30

**Authors:** Elena-Cristina Preda, Oana Oprea, Zsolt Albert Barabas, Ioana Paula Simion, Ana-Maria Fotache, Minodora Dobreanu

**Affiliations:** George Emil Palade University of Medicine Pharmacy Science and Technology of Targu Mures,Romania

**Keywords:** creatinine, glomerular filtration rate, clinical decision-making, renal function tests

## Abstract

**Background:**

Accurate measurement of serum creatinine (SCr) is critical in estimating glomerular filtration rate (eGFR) and classifying kidney function. This study evaluated the analytical differences between the enzymatic and Jaffe methods for SCr measurement and their impact on eGFR estimation using two widely applied equations: CKD-EPI and EKFC.

**Methods:**

The study included 427 patients over 40 years old. SCr was measured using both enzymatic and Jaffe methods on the Alinity c platform. eGFR was calculated with the CKD-EPI (2009) and EKFC equations. Agreement between methods was assessed using Bland-Altman and Passing-Bablok regression. eGFR differences were analyzed using the Wilcoxon signed-rank test and multiple linear regression. Agreement in GFR category classification was evaluated using weighted kappa and Kendall’s tau.

**Results:**

While the mean difference between methods was small, both systematic and proportional biases were statistically significant. eGFR values differed significantly between methods in both sexes (p < 0.01), regardless of the equation used. ΔeGFR was significantly associated with SCr values, but not with age. Although overall agreement in GFR categories was high (kappa > 0.91), method-dependent reclassification of patients was observed, which may influence CKD diagnosis and clinical decision-making.

**Conclusions:**

Even minor analytical differences between enzymatic and Jaffe SCr measurements can lead to clinically relevant discrepancies in GFR categorization. These findings highlight the need for harmonization in laboratory methods to ensure consistent reporting and patient management.

## Introduction

Creatinine, the end product of creatine and creatine phosphate metabolism [1], is the most commonly used biomarker of kidney function. Serum creatinine (SCr) is routinely used to estimate glomerular filtration rate (eGFR), which forms the basis for the diagnosis and staging of chronic kidney disease (CKD) [2] and it is also considered one of the main criteria for the diagnosis of acute kidney injury (AKI) [3].

Renal function assessment plays a key role in clinical decision-making, risk stratification, and drug dosing across various medical specialties [4]. Age is also an important factor, as renal function begins to physiologically decline after the age of 40, making the estimation of GFR particularly relevant in the management of older patients [5].

Considering its clinical importance, the SCr is widely measured in laboratories. There are two methods frequently utilized: Jaffe and enzymatic. In the Jaffe method, the creatinine reacts with alkaline sodium picrate, resulting in an orange complex [6]. The enzymatic method uses a series of enzymes to convert creatinine into compounds that generate color, enabling measurement of serum creatinine. These reactions produce detectable substances such as ammonia or sarcosine [7]. The main substances that interfere with the Jaffe method include high protein levels, hemoglobin from hemolysis, bilirubin, lipids, glucose, ketone bodies, ascorbic acid, and certain medications, with such interferences being less common with enzymatic method [8].

However, in Europe, approximately 57% of creatinine measurements are performed using the Jaffe method, due to its cost-effectiveness compared to other techniques [9], while enzymatic method is used in 39% of the laboratories [10]. eGFR can be calculated using different creatinine-based equations, which may differ in their performance across age ranges and clinical settings. The Chronic Kidney Disease Epidemiology Collaboration 2009 (CKD-EPI) equation remains widely used in routine practice [11], while the more recent European Kidney Function Consortium (EKFC) equation has been recently proposed for the full adult age spectrum [12].

The aim of this study was to compare enzymatic and Jaffe serum creatinine measurement methods and to assess their impact on eGFR estimation and GFR category classification using the CKD-EPI and EKFC equations in patients over 40 years of age.

## Material and Methods

This study was conducted during the month of July 2024 at the Clinical County Emergency Hospital of Târgu Mureș, Romania. Ethical approval was obtained from the local institutional ethics committee (No. 12861/28-05-2024).

This study was performed using anonymized left-over serum samples collected for routine clinical testing, from patients over 40 years old. The samples were selected across the analytical range. Samples with hemolysis, lipemia, or icterus were excluded according to laboratory quality criteria.

Serum creatinine (SCr) was measured using the two methods available on the Alinity c analyzer (Abbott Laboratories, USA). Both the enzymatic and Jaffe creatinine assays were calibrated according to the manufacturer’s specifications and were traceable to isotope dilution mass spectrometry (IDMS) reference methods. The enzymatic method was considered as comparative analytical reference, due to its known accuracy. eGFR was computed using two equations: 2009 CKD-EPI [11] and EKFC [12].

### Method Comparison

To evaluate the agreement between the enzymatic and Jaffe methods, the Bland-Altman analysis (to assess the mean difference and limits of agreement) and the Passing-Bablok regression were performed according to CLSI guidelines for method comparison [13]. Analyses were conducted both on all samples included and on a subset of samples with SCr < 1.5 mg/dL, specifically addressing the range where SCr has reduced sensitivity for detecting renal impairment [14] and it is a commonly used clinical cutoff for contrast imaging [15].

### Impact on eGFR

The effect of the creatinine measurement method on eGFR values was evaluated with Wilcoxon signed-rank test, by comparing the eGFR derived from SCr measured with enzymatic method (SCr_ENZ_) versus SCr measured with Jaffe method (SCr_Jaffe_).

To highlight the differences of SCr method determination on the eGFR, we calculated the difference between eGFR values obtained using the enzymatic and Jaffe methods (ΔeGFR) and applied a multiple regression model with Scr_Jaffe_, SCr_Enz_ values, and age as independent variables. Since age is a component of the eGFR estimation equations, it was included as an independent variable to evaluate whether ΔeGFR vary with increasing patient age.


$$\Delta \text{eGFR}=\text{eGFR}\;\text{derived}\;\text{from}\;{\text{SCr}}_{\text{ENZ}}-\text{eGFR}\;\text{derived}\;\text{from}\;{\text{SCr}}_{\text{Jaffe}}.$$

### eGFR Category Reclassification

Patients were classified into eGFR categories [16] based on each SCr determination method and for each eGFR equation. The agreement between classification results was evaluated using Kendall’s tau (for ordinal correlation) and weighted kappa (for inter-rater agreement). Tau coefficient (τ) and kappa were interpreted according to Schober et al. [17] and Landis et al. [18], respectively.

Statistical analyses were performed using MedCalc (MedCalc Software Ltd, Belgium) and IBM SPSS Statistics, version 20. Statistical significance was defined as p < 0.05. Separate analyses were performed for male and female patients to assess the impact of the SCr measurement method on eGFR values and the subsequent reclassification into eGFR categories.

The term under-classification refers to assigning patients to a more severe GFR stage, while up-classification refers to reassigning patients to a milder GFR stage. Discordant cases refer to patients who are categorized into different GFR stages depending on the creatinine measurement method.

## Results

A total of 427 patients were included, out of which 213 were females and 214 males. The age range was 42 to 98 years, with a median of 67 years. Patients came from different hospital departments. Acute and critical care settings included the intensive care unit and emergency department. Medical wards included cardiology, internal medicine, gastroenterology, hematology, and neurology. Surgical wards included general surgery, vascular surgery, oral and maxillofacial surgery, orthopedics, and neurosurgery. Chronic care specialties included nephrology, diabetology, and rheumatology. Approximately 30% of patients were from acute and critical care settings, 49% from medical wards, 11% from surgical wards, and 10% from chronic care specialties.

Serum creatinine values ranged from 0.66 to 18.59 mg/dL (enzymatic) and 0.66 to 19.20 mg/dL (Jaffe). The distribution of serum creatinine (SCr) values and demographic characteristics is summarized in Table 1. According to Kolmogorov-Smirnov test, most variables presented in Table 1 had non-gaussian distribution, the only exception with normal distribution was the age in male patients.

**Table 1.** Descriptive statistics for patient age and serum creatinine values measured by enzymatic and Jaffe methods.

**Variable:**	**Age**	**Female age**	**Men age**	**SCr_Enzymatic_ (mg/dL)**	**SCr_Jaffe_ (mg/dL)**
Number	427	213	214	427	427
Min	42	42	42	0.66	0.66
Max	98	98	90	18.59	19.2
Mean/Median	67	66	66	1.78	1.79
SD / IQR	15	6.25	11.6	1.7	2.2

Values are reported as number of observations, minimum and maximum, and either mean ± standard deviation (SD) or median and interquartile range (IQR), depending on the distribution of each variable.

### Comparison between creatinine methods

**Fig. 1.**
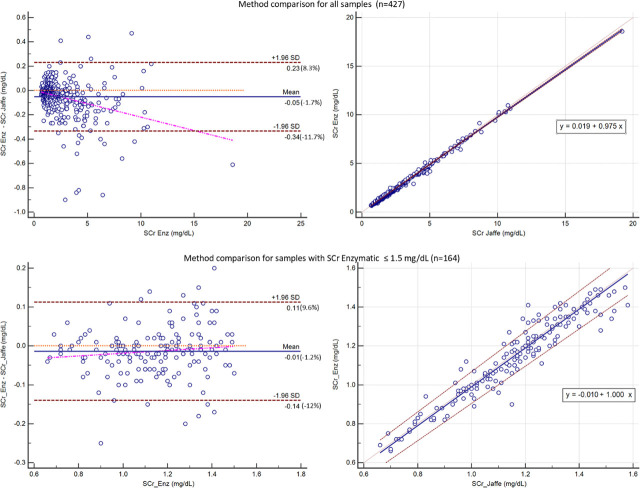
Comparison between enzymatic and Jaffe methods for serum creatinine (SCr) measurement. The upper part of the figure shows the analysis for all samples, while the lower part shows the analysis for samples with SCr_ENZ_ < 1.5 mg/dL. Bland-Altman plots are shown on the left, and Passing-Bablok regression plots on the right.

When comparing the two methods across the full range of our data set, the Bland Altman analysis showed no significant mean difference between the enzymatic and Jaffe methods (1.7%). However, Passing-Bablok regression indicated that both systematic bias (intercept = 0.019, 95% CI: 0.006 to 0.030) and proportional bias (slope = 0.975, 95% CI: 0.969 to 0.981) were statistically significant, with no evidence of deviation from linearity. (See Figure 1)

In the subset of SCr <1.5 mg/dL, the differences between methods were minimal, with no statistical significance (1.2%). The intercept was −0.01 (95% CI: −0.055 to 0.033) and the slope was 1 (95% CI: 0.959 to 1.036). (See Figure 1)

### Impact of SCr_Jaffe_ on eGFR Values

There was a statistically significant difference between eGFR values calculated using *SCr_ENZ_* and those calculated using *SCr_Jaffe_*. This difference was observed for both the CKD-EPI and EKFC equations, in both male and female patients, with the Wilcoxon analysis returning p<0.01 in all cases.

Linear regression analyses demonstrated that ΔeGFR was significantly associated with both creatinine values. Age had no statistically significant effect in either sex. Results are shown in Table 2.

**Table 2.** Multiple linear regression models for ΔeGFR by sex and eGFR equation (CKD-EPI, EKFC), with SCr (enzymatic, Jaffe) and age as predictors.

**Sex**	**Equation**	**Predictor**	**B**	**p**
Male	ΔeGFR_CKD-EPI_	SCr_Enz_	−11.579	<0.001
		SCr_Jaffe_	11.17	<0.001
		Age	0.005	0.756
	ΔeGFR_EKFC_	SCr_Enz_	−11.042	<0.001
		SCr_Jaffe_	10.667	<0.001
		Age	0.004	0.783

Female	ΔeGFR_CKD-EPI_	SCr_Enz_	−8.497	<0.001
		SCr_Jaffe_	8.235	<0.001
		Age	−0.018	0.153
	ΔeGFR_EKFC_	SCr_Enz_	−7.684	<0.001
		SCr_Jaffe_	7.443	<0.001
		Age	−0.019	0.097

ΔeGFR - eGFR based on SCr_ENZ_ minus eGFR based on SCr_Jaffe_. B = unstandardized regression coefficient. CKD-EPI = Chronic Kidney Disease Epidemiology Collaboration; EKFC = European Kidney Function Consortium.

**Table 3.** Agreement in eGFR-based CKD staging between enzymatic and Jaffe methods using CKD-EPI and EKFC equations, assessed by weighted kappa and Kendall’s tau, stratified by sex.

**Equation**	**Male patients**		**Female patients**	
	**Weighted Kappa**	**Kendall’s Tau**	**Weighted Kappa**	**Kendall’s Tau**
CKD-EPI	0.928 (CI: 0.900–0.956)	0.949	0.923 (CI: 0.894–0.951)	0.943
EKFC	0.916 (CI: 0.885–0.947)	0.95	0.943 (CI: 0.918–0.969)	0.959

## eGFR Category agreement and reclassification

Agreement between GFR classification based on eGFR values derived from Enzymatic and Jaffe methods for SCr was high (See table 3), but differences still resulted in patient reclassification. When using CKD-EPI, a total of 10.77% discordant cases were found and 10.07% when using EKFC.

**Fig. 2.**
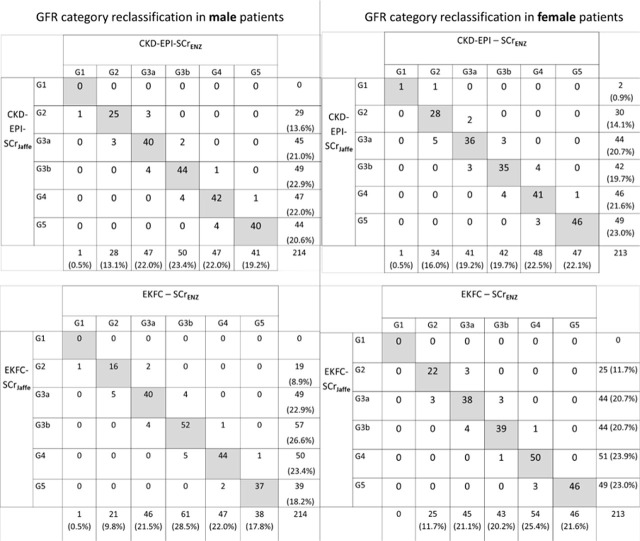
Reclassification matrix of GFR categories in male (left side) and female (right side) patients using CKD-EPI (upper side) and EKFC (down) equations using enzymatic and Jaffe serum creatinine measurement.

Analyzing the reclassification for each sex, the method of measurement of SCr resulted in the reclassification of 8.88% males and 12.20% female patients when the CKD-EPI was used, and 11.68% males and 7.45% females when using EKFC. For both equations, the use of SCr_Jaffe_ led to a minor but constant trend of under-classification, moving patients to a more severe GFR stage. (See Figure 2)

## Discussion

This study highlights that the method used to measure SCr can have an impact on the estimation of GFR and the classification into GFR categories. Even small differences in creatinine values can be clinically relevant for the patient diagnosis and management. Our study focused on patients over 40 years old, a threshold known for the beginning of physiological decline in renal function [5, 19].

### Method comparison between enzymatic and Jaffe methods

In our study, the comparison between enzymatic and Jaffe methods revealed that the two methods are highly comparable at lower SCr levels (< 1.5 mg/dL), as indicated by the minimal mean difference and nearly perfect agreement in regression analysis. This high level of agreement at normal and moderately increased SCr levels is relevant for screening and early detection of CKD, where small inaccuracies in creatinine measurement can lead to significant misclassification of patients, especially in the “blind zone” of creatinine, where early functional decline may not be reflected by substantial increases in SCr [20]. However, as SCr concentrations increase, the differences between the two methods become more important.

When analyzing the full range of SCr in our dataset, Bland-Altman analysis showed good agreement between the enzymatic and Jaffe methods. Passing-Bablok regression revealed both systematic (0.02 mg/dL) and proportional bias (0.98), indicating that Jaffe measurements tend to be higher than enzymatic ones across the range. Similar results were obtained in a study evaluating the differences between Jaffe and Enzymatic methods for SCr on Abbott Architect [21] or other platforms [22]. In another study also on Architect analyzer no systematic differences were between the methods [23].

Although the average difference between enzymatic and Jaffe creatinine measurements was small (−1.7%), the relative limits of agreement were wide, reaching from 8% to −12% in the whole cohort, with similar ranges in samples with SCr < 1.5 mg/dL. The European Biological Variation Study (EuBIVAS) report a within-subject biological variation (CVi) for serum creatinine of 4.4%, indicating that small changes in creatinine may fall within expected biological variation [24]. In this context, the width of the between-method limits of agreement suggests that analytical differences may approach or exceed normal CVi.

### Impact of SCr measurement method on eGFR

Considering the inverse relationship between SCr and eGFR, a method that overestimates the SCr value will consequently result in an underestimation of eGFR. Although small, these between-method differences did translate into significant differences in eGFR calculation with both equations. Significantly lower eGFR values were obtained when using SCr_Jaff_ with both CKD-EPI and EKFC equations. This effect was consistent, regardless of patient’s sex, showing a trend of underestimation of the GFR when Jaffe method is used.

Regression analysis of ΔeGFR indicated that the creatinine assay method was the main factor causing discrepancies. The B coefficient had almost identical values for each eGFR equation used, for both SCr_Jaffe_ and SCr_Enz_, and for each sex. The positive B values for Scr_Jaffe_ and negative B values for SCr_Enz_, revealed that the ΔeGFR decreases as the SCr_Enz_ values increase and vice versa for SCr_Jaffe_. Therefore, the Jaffe method tended to underestimate renal function, while the enzymatic method, known for its higher accuracy, returned higher eGFR values. Growing in age had no significant impact on the ΔeGFR, emphasizing the role of the analytical method of SCr as the primary determinant.

### eGFR Category agreement and reclassification

The ordinal classification of GFR categories was high (τ > 0.94) and also the agreement between eGFR equations using SCr_Jaffe_ and SCr_Enz_ was also very good (kappa >0.91). Despite this high agreement, a proportion of approximately 10% of patients were reclassified when using CKD-EPI or EKFC. Regardless of eGFR equation of choice, discordant cases included both up-classification and under-classification. However, a trend of under-classification of patients when eGFR was calculated using SCr_Jaffe_ was still observed.

Similar reclassification pattern was described by Syme et al, when using the 2009 CKD-EPI equations [21]. In a study on a cohort of diabetic patients, high kappa agreement of 0.92 was found, with similar percentages (9%) of discordant cases [25]. In that study, 8% of the discordant cases were under-classified using Jaffe method, while in our study between 5.2 and 7.9% were also under-classified [25]. Another study in Germany, with over 12,000 pairs of SCr measurements from outpatient kidney transplant recipients, the Kappa agreement was 0.84 for EKFC equations and 0.83 for CKD-EPI [26].

From a clinical perspective, the greatest sensitivity to eGFR-based decision-making occurs in the intermediate GFR stages, while small variations in eGFR are less likely to influence clinical decisions in patients with preserved renal function (G1–G2) or advanced kidney failure (G5). The G3a, G3b and G4 categories require dose adjustment or treatment review for many commonly used medications. The KDIGO guidelines for CKD and AKI recommend to rely on GFR categories rather than continuous values for medication such as metformin, direct oral anticoagulants, renally cleared antibiotics and non-steroidal anti-inflammatory drugs [2, 3]. In these situations, method-dependent reclassification across GFR categories, as observed in our study, may influence patient management when values are close to decision thresholds.

A similar approach applies to the use of iodinated contrast media, where eGFR is used to guide risk stratification and preventive measures for contrast-induced AKI, particularly around the 30 mL/min/1.73 m^2^ threshold [27].

Although method-dependent reclassification was observed, overall agreement between enzymatic and Jaffe methods remained high. Therefore, the present findings should not be interpreted as evidence of systematic diagnostic error, but rather as an illustration of how analytical variability may become clinically relevant when eGFR values are close to medical decision levels.

This study has a few important limitations to consider. First, measured GFR, the gold standard for assessing kidney function, was not determined. Second, the study did not include in the regression model other influencing factors such medication or protein levels. Third, measurements were performed on a single analytical platform, which may limit generalizability.

## Conclusion

The results of our study showed that small but systematic analytical differences between enzymatic and Jaffe serum creatinine methods translate into statistically significant differences in eGFR values and may result in reclassification across clinically relevant GFR categories. Despite the overall good agreement, such differences may become relevant in acute and critical care settings, where decisions are frequently guided by GFR categories. Recognizing and accounting for between-method differences is essential for the accurate evaluation and monitoring of renal function.
